# The evolutionary entanglement of flipons with zinc fingers and retroelements has engendered a large family of Z-DNA and G-quadruplex binding proteins

**DOI:** 10.1098/rsob.250171

**Published:** 2025-10-22

**Authors:** Alan Herbert

**Affiliations:** ^1^ Department of Discovery, InsideOutBio Inc., Charlestown, MA, USA

**Keywords:** flipons, zinc-finger proteins, Z-DNA, G-quadruplex, transcription, topological domains, hybrid sterility, noncoding RNA, DNA methylation, Mendelian disease

## Abstract

Sequences called flipons can adopt discrete, alternative nucleic acid conformations, such as the left-handed Z-DNA and Z-RNA double helices (referred to collectively as ZNA), and the four-stranded RNA and DNA G-quadruplexes. Each flipon conformation encodes different information. For example, the base-specific interactions of proteins with B-DNA enable sequence-specific recognition. In contrast, the higher energy Z-DNA and G-quadruplexes facilitate the speedy scanning of chromosomes to locate active regions of the genome. Results synthesized from small-scale benchside and large-scale computational experimental approaches provide compelling evidence that zinc-finger protein domains (ZFDs) not only engage in base-specific recognition of B-DNA, but also bind directly to Z-DNA and G-quadruplexes. The findings address the long-standing speed–stability paradox of how high-affinity ZFPs with multiple zinc fingers can rapidly localize to a specific binding site. The energy gap between different DNA interaction modes enables fast off-rates during the scanning of Z-DNA for cognate binding sites, and a slow off-rate following engagement of the B-DNA conformer. ZFPs represent the most prominent human transcription factor family with 804 annotated members. The coevolution of flipons and ZFP enhances suppression of retroelements and enables rapid, context-specific responses. ZNA and GQ binding proteins are consequently more frequent in the proteome than currently conceded.

**Figure:**
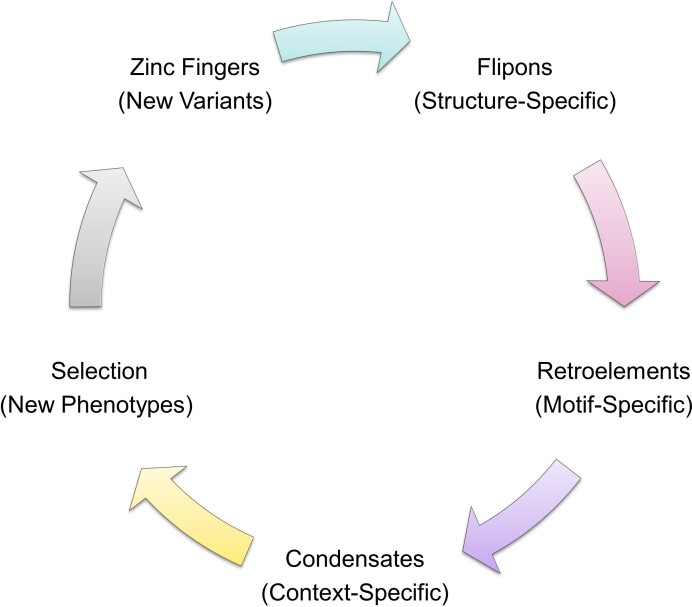

## 1. Introduction

Recent discoveries have revealed the encoding of information in DNA by both structure and sequence. DNA conformers other than the right-handed B-DNA can form under physiological conditions and alter cellular responses without base-specific recognition of the nucleotide sequence. These alternative conformations include the left-handed Z-DNA duplex, the three-stranded triplex, the four-stranded quadruplex and the i-motif formed by the intercalation of cytosine base pairs. Each non-B-DNA structure is encoded by genetic elements named flipons [1]. Each flipon type has a canonical repeat motif and is subject to natural selection. The spread of flipon motifs throughout the genome is driven by transposable elements (TEs) that rely on flipons to regulate their expression and insertion into new chromosomal loci [2]. TEs also drive the expansion of zinc-finger protein (ZFP) [3–5]. Those ZFPs with N-terminal Krüppel-associated box effector domains suppress TEs in a sequence-specific manner. Here, the connection between flipons and zinc-finger evolution is synthesized using data from small-scale benchside and large-scale computational experimental approaches. The analysis explores the intimate connections between flipons and ZFP evolution.

## 2. Repeats and flipons

The human genome is primarily composed of repeat elements. Depending on the algorithm used, between 45% and 63% of the nucleotide sequence derives from mobile TEs [6]. The telomere-to-telomere sequencing consortium annotation of the pseudo-haploid human cell line CHM13 reported that long interspersed nuclear elements (LINES) contributed 21.34%, short interspersed nuclear elements (SINES) 13.22% and long terminal repeats (LTRs) that include endogenous retroviruses (ERVs) 9.15% of the sequence [7]. Other repeat elements can expand or contract through replication or repair errors, or by recombination, including those with DNA dinucleotide and variable number of tandem repeats (VNTRs). DNA transposons and more complex repeats such as SINE-VNTR-Alu (SVA) contribute 3.84% of the sequence. The same accumulation of repeat elements is found in many other metazoans [6].

The high frequency of repeats in the genome renders them uninformative, as targeting each individually is challenging. However, it is possible to target the subset if repeats that can adopt an alternative conformation when there is sufficient available energy to initiate the transition. This energy is produced in active parts of the genome by transactions involving helicases, polymerases and the eviction of histones by chromatin remodellers. The repeats that change conformation under physiological conditions are called flipons. The alternative structures formed include left-handed Z-DNA with its canonical d(CG)_n_ motif [8,9], three-stranded triplexes that have a d(A/G)_*n*_ repeat [10], four-stranded G-quadruplexes formed by four repeats of a d(G)_*n*>2_ repeat [11], and i-motifs in which cytosines base-pair to each other, intercalate to form a tetrad [12]. The flip from B-DNA to these alternative structures involves rotation of nucleobases around the glycosidic bond. In B-DNA, all the bases point away from the sugars in the anti-position, while in other structures, one or more of the base lies over the sugar in the syn-conformation. The exact number of anti- and syn-bases, and whether the base-pairing involves Watson–Crick or Hoogsteen hydrogen-bonding, depends on the flipon type.

Flipons enable the structure-specific assembly of different complexes at particular sites in a cell [13–22]. During the process, different proteins cooperate to cycle flipons from one conformation to another. The structure-specific binding of the Zα domain to Z-DNA and Z-RNA (collectively called ZNAs) stabilizes the left-handed conformation [23–26]. The interaction does not involve any base-specific contacts. The complex is stable due to the slow off-rate of Zα from ZNA. The flipons bound play crucial roles in regulating innate immune responses to viruses like influenza, smallpox, measles and SARS, and to retroelements embedded in the genome [27–34]. Both the Zα domain and the flipons bound are subject to selection by pathogens. The epidemics occurring during the rapid urbanization of human populations were highly lethal, as demonstrated by their effects on previously unexposed individuals in the New World. The only two proteins with Zα domains in the human genome, ADAR and ZBP1, protected against measles, smallpox, influenza and, more recently, against SARS-CoV-2 [35–43].

A domain related to Zα in transcription factor E also plays a role in resetting promoters to enable another round of transcription [44]. Several proteins, such as topoisomerases, helicases and chromatin remodellers, also modulate Z-DNA formation [45–48]. Different sets of proteins temper G-quadruplex cycles and play essential roles in transcription, DNA replication, RNA processing and translation regulation [49,50]. Triplexes are another flipon conformation that helps define the boundaries of nucleosome-free regions and the transcripts produced by a cell. The processes involved in triplex formation represent a collaboration between proteins, DNA and RNA, leading to the nucleation of condensates that bring enhancer and promoter elements together. The interactions result in the assembly of cellular machines that architect the three-dimensional structure of chromatin [51–55].

## 3. Coding with flipons

By changing structure, flipons play an essential role in these various cellular transactions by altering how information is read from the genome. Many different RNA transcripts can be produced from a gene by alternative splicing and RNA editing of the primary transcript [56]. The outcome depends on the protein complexes assembled at each locus in the genome. The nature of the proteins bound varies with the flipon conformation. As a consequence, the transcripts produced can change dynamically, without altering the DNA sequence or breaking either DNA strand. In the nucleus, flipons play roles in setting promoter usage, RNA splicing and editing, and transcript termination. In the cytoplasm, the impact is on transcript stability, translation and the cellular scaffolds formed [19,20,57–59]. These properties are exploited by retroelements, which spread by copying their transcripts into DNA and inserting these regulatory elements into new genomic sites.

SINE repeats are the ultimate abstraction of the flipon coding potential [2]. These non-autonomous retrotransposons do not code for protein [60]. Instead, they must recruit the transcriptional machinery necessary to produce RNA, then capture reverse transcriptases encoded by either LINEs or LTRs to ensure the transcripts are copied back into the genome as DNA. Flipons encoded by SINE sequences actuate these processes by recruiting the transcription factors and other protein complexes necessary to complete each step. For example, SINE-encoded triplexes position nucleosomes to align enhancers and promoters in chromatin loops to promote their expression [55], while SINE-encoded G-quadruplexes facilitate their recruitment to ribosomes to capture a rverse transcriptase the instant it is produced.

The replication of SINEs is limited by the Zα domain proteins adenosine deaminase RNA-specific (ADAR) and Z-DNA binding protein (ZBP1). The ZNAs encoded by SINEs [61] activate ZBP1 to induce cell death when retrotransposition is active [41,47,62–66]. At the same time, ADAR negatively regulates retrotransposition by adenosine-to-inosine editing of SINE transcripts to promote their degradation. Notably, millions of the edits found in cellular mRNAs are in inverted repeat SINE elements that fold as double-stranded RNAs. These edits impact the mRNA half-life [67–70]. By binding Z-RNA, ADAR also prevents normal cellular RNAs containing SINE-sequence inserts from activating ZBP. The threshold set by ADAR is overcome by the 100 or more fold induction of ZBP1 by interferon during infections, and by the interferon stimulated expression of extragenic and intronic ERE-derived Z-RNAs.

LINES, ERVs and LTRs also contain flipons within their promoters. GQs are present in the conserved 5′-LTR U3 region of lentiviruses, while LINE L1 elements form GQs in their 3′-UTR [71–73]. Z-flipons are also present in LINE-1 promoters [29,66,74]. Further, TPXs present in LINE-L1 have important roles during development [75]. These retroelements frequently undergo homologous recombination, either between the 5′- and 3′-LTRs derived from each other during insertion, or with other retroelements from the same family [76,77]. The events produce solitary (solo) LTRs that cannot encode any of the proteins required for retrotransposition. Like SINES, these solo LTRs abstract flipon sequences involved in gene regulation. These flipons undergo selection and contribute to the complexity of cellular programmes.

## 4. Targeting of transposable elements by small RNAs and zinc-finger proteins

Small RNAs, including microRNAs and piRNAs, evolved early in a broad range of species to suppress LINES, LTRs and ERVs in [78,79]. ZFPs provided another way for hosts to target TEs. The ZFP arose early in eukaryote evolution, before the split between protists, plants, and animals [80–82]. ZFP subsequently evolved into transcription factors as ERE elements were exapted to regulate transcription of cellular genes. Of the 1639 curated transcription factors in animals, around 747 are ZFPs, where the zinc ion is coordinated by two cysteines and two histidines (C_2_H_2_ family) [82]. Around 712 have multiple tandem C_2_H_2_ zinc finger domains [83]. They are encoded in humans by 423 loci [84]. Another 57 C_3_H_1_-type ZFPs are also annotated [85].

The C_2_H_2_-ZFP motif is relatively simple. The two histidines are in an α helix and coordinate with two cysteines present in a β-hairpin, in which two strands fold back on each other to stabilize the structure. The domain consists of 23−30 amino acids with a (F/Y)-X-C-X2−5-C-X3-(F/Y)-X5-ψ-X2-H-X3−5 H motif, where X is an amino acid and ψ is a hydrophobic residue [86,87]. The simplicity of this fold and the early evolutionary origins connect these domains with the tinkers that are thought to drive the initial amplification of peptide–nucleic complexes, with metals acting as catalysts in their replication [88].

Pairing of ZFP with different effector domains allows the localization of protein complexes that can modulate gene expression in various ways. Examples include the Krüppel associated box (KRAB), SCAN (SRE-ZBP, CTfin51, AW-1 and Number18 cDNA), the poxvirus and zinc finger (POZ) domain that is alternatively named as the BTB domain (Broad-Complex, Tramtrack and Bric-a-brac), and DUF3669 (Domain of Unknown Function 3669) domain at their N-termini [81,89]. An analysis of Krüppel-like factor and specificity protein (KLF/SP) genes across 48 Eukarya species reveals the existence of many more effector domains [90]. The KRAB recruits Chromobox protein homologue 5 (CBX5, heterochromatin protein 1 (HP1) and the SET domain bifurcated histone lysine methyltransferase 1 (SETDB1), catalysing deposition of the repressive histone H3 lysine 9 trimethyl (H3K9me3) mark [91].

## 5. Zinc-finger protein and effector domains

Of the 747 human C2H2-ZFP, as many as 377 contain a KRAB domain, and another 28 that have both KRAB and SCAN domains [82,84,92,93]. The KRAB domain proteins contain a median of 11 separate ZFs, ranging from 3 to 38 copies, with the potential to bind sequences 9–114 nucleotides in length [83]. Usually, the ZFs are encoded by a separate exon [94]. In tetrapods, the rapid increase and diversification of KRAB-ZFP (KZFP) is correlated with the expansion of TE [5,81,91,94–100].

The coevolution of KZFP and the retroelements they target has led to the concept of an ‘evolutionary arms race’ [5]. ZFPs emerge to suppress retrotransposons, which escape by the deletion or mutation of the target sequences. The TEs may lose sequence blocks as large as 129 nucleotides [5], or avoid censure by a single base mutation [100]. ZFPs then evolve to counter this new threat [100]. Those KZFPs that target the internal protein-coding regions of the TEs [100] likely exist only to suppress TEs capable of retrotransposition.

However, not all TEs in the human genome are recognized by KZFP. By one estimate based on chromatin-immunoprecipitation studies, TEs only represent two-thirds of sequences bound by KZFPs. Other targets include promoters and simple repeats. Additionally, many of the TEs that KZFPs bind are incapable of retrotransposition [4,81]. Notably, only a small fraction of TEs are engaged by KZFPs [81,97]. In contrast, new TEs can be targeted by pre-existing KZFPs, especially those like ZFP57 that are involved in the parental imprinting of genes [101]. Recognition of other newly evolved TEs is facilitated by six major clusters of KZFP on chromosome 19 that are hotspots for copy number variation. The heptamer sequences 3′ to those that specify the Zn-binding histidines resemble those used in immunoglobulin gene rearrangement and may contribute to intralocus recombination [21]. RNA editing and intergenic alternative splicing based on a heptamer repeat may further expand the variants generated. The recombinants and variants generated are then subject to intense selective pressure. Those individuals unable to achieve control of TE expansion are less likely to produce multigenerational progeny [102]. The form of selection is likely rapid, faster than the 7 million years previously estimated [4].

## 6. Binding specificity of KZFP

Traditionally, KZFPs have been classed as sequence-specific B-DNA binding proteins due to the nature of the base contacts they make. The initial crystal structures led to a proposal that each ZFP binds specifically to a 3-nucleotide sequence through amino acids at positions −1, 2, 3 and 6 of the ZFP α-helix that were termed ‘specificity residues’ [86,87]. This finding was evaluated by a high-throughput analysis of around 20% of eukaryotic C_2_H_2_-ZFP domains incorporated into a three-ZFP module. The authors estimated that overall, such modules could recognize approximately 450 distinct DNA motifs. Surprisingly, those motifs derived from KZFP were not enriched in ERE sequences relative to non-KRAB-associated ZFPs [103].

Another systematic survey of bonding specificity was based on synthetic ZFPs made by re-engineering Zif268. All 20 possible amino acids in the −1, 1, 2, 3, 5 and 6 positions of the middle or C-terminal ZFP α-helices were screened against all 64 possible 3-nucleotide sequences to relate amino acid sequence to nucleotide specificity [104]. The total number of ZFPs assessed far exceeded those present in biological systems. Jointly, the two studies revealed that the binding motif of a ZFP is affected by non-specificity residues in each domain and by interactions between neighbouring ZFP domains. Further, there was degeneracy, with many 3-nucleotide sequences bound by hundreds to thousands of different ZFP domains with unrelated amino acid specificity residues [104].

A structural analysis revealed that neighbouring ZF domains affect the binding mode, leading to the recognition by each ZFP of different nucleotides. The six modes (four supported by experimental data) covary with amino acids at positions −2 and +9 in the interface between the ZF domains [105]. Collectively, the work enabled the development of improved algorithms to predict the specificity of a particular ZFP amino acid sequence, inspired by earlier methods developed with much more limited datasets [106]. Newer models also incorporate structural information from crystals, with accuracy varying from 60% to 90%, and dependent on the experimentally determined motif tested [107].

An analysis of ZFP from 283 eukaryotic genomes revealed remarkable differences in the evolution of ZFPs between non-metazoans and metazoans [108]. Notably, the last common ancestor likely recognized all possible DNA triplet sequences. Non-metazoan ZFs now recognize only a small subset of possible DNA triplets. The sequences have evolved more slowly compared to metazoans and are fewer in number. Most of the binding specificity involves base-specific contacts. Metazoan C_2_H_2_-ZFs recognize a diverse set of sequences and are predicted to recognize all possible DNA triplet sequences. The binding affinity depends on DNA backbone contacts that compensate for the lower specificity of base interactions. As a consequence, the affinity of binding can be maintained as single-base mutations drastically alter the nucleotide motif bound [108].

Other studies of ZFP in yeast also revealed that ZFPs could acquire additional specificity for other sequences without losing affinity for those motifs bound in common with other family members. The acquired function reflected amino acid changes outside the recognition helix, and variations in non-specificity residues within the helix, either in a single ZF domain or combined with others in an adjacent ZF [109]. A similar process also evolves the bispecificity of forkhead transcription factors [110]. Further adaptability arises as ZFP arrays can recognize different consensus sequences through separate ZF domains [111].

The duplication of ZFPs during genome evolution also adds to an organism’s pliability. In yeast, the Multicopy Suppressor of SNF1 mutation 2 (MSN2) gene regulates robust gene expression under basal conditions with only slight variation in levels. The paralogue MSN4 is induced only under stress conditions. Natural selection optimizes the expression level to counter such perturbation, producing little noise in the response [112]. These different mechanisms collectively are optimized during ZFP evolution. Protein variability is tolerated and can produce a gain-of-function while maintaining existing interactions. The design reduces the risk that a single nucleotide mutation produces an adverse outcome, while increasing the sequence space available for exploration.

ZF domains also engage in other interactions besides those with DNA. They also bind RNA [113]. Other ZF domains do not bind nucleic acids at all. Instead, they serve as protein interaction domains [114–116]. In the following sections, I provide evidence that ZFPs also engage different flipon conformations. These transactions enhance the functionality of ZFPs even further. This relationship is quite ancient, with flipons and ZFPs still coevolving. The entanglement traces back to the early origins of both ZFPs and flipons and their interactions with TEs. The result is the retention and expansion of both genetic elements in the genomes we know today [80,117].

## 7. Zinc-finger protein and flipons

While it is natural to map protein binding to a single DNA conformation, many examples exist where proteins bind different nucleic acid structures. Aside from ZFP, many proteins bind both B-DNA and A-RNA [118]. Experimentally, transcription factors, like the yeast Repressor Activator Protein (RAP1), can bind to B-DNA and GQ through the same domain [119,120]. Likewise, there are flipon sequences that can adopt alternative folds to bind different sets of proteins. For example, d(G)_*n*_ repeats can form hairpins, a fold-back G-triplex structure, GQ, a multi-strand TPX and Z-DNA, depending on the context [55,121–132].

Certainly, in modern genomes, flipons help maintain open chromatin regions that enable the binding of ZFP and other TF to their cognate sequences [55,133–136]. Further, the recognition of flipon structures by ZFP allows the delivery of effector complexes to a specific locus in the genome. These targeted interactions allow modulation of gene expression in a context-specific manner. Currently, experimental evidence supports the binding of ZFP proteins to B-DNA and GQ. An example is provided by the transcription factor specificity protein 1 (SP1), which binds sequence-specifically to B-DNA and to the GQ structure [137,138]. The stability of the GQ fold, and the dependence on nucleotides able to Hoogsteen base pair, enabled the discovery of this binding mode. However, identifying dual binding modes for less stable flipon structures like Z-DNA is more challenging, given that the interaction with the higher energy left-handed DNA is likely to be transient.

**Figure 1.**
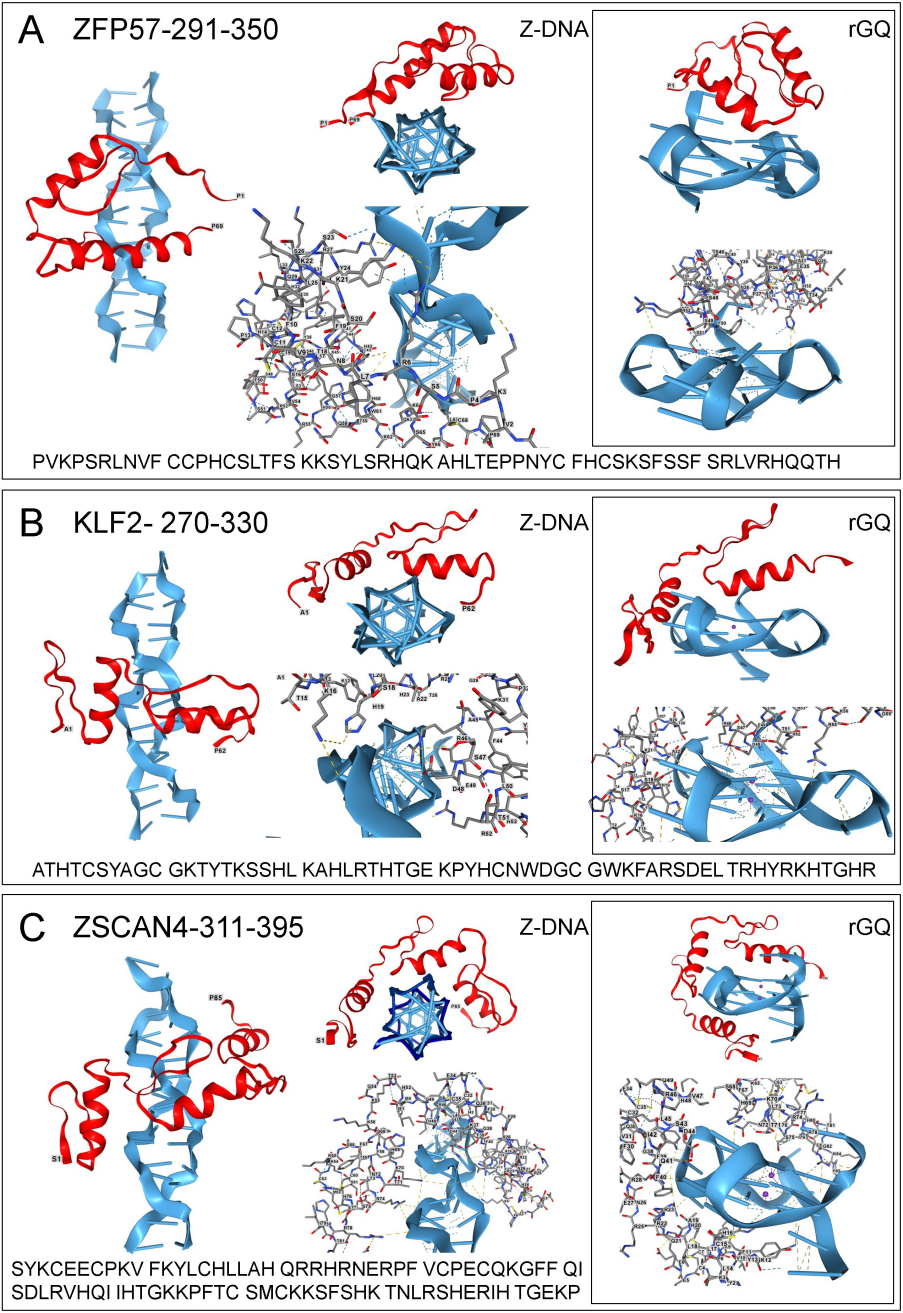
Docking of Krüppel Box associated ZFPs to Z-DNA and RNA. Z-DNA and GQ are coloured blue, and the ZFP is ruby. Each panel has views of the structure from the side and the top, and also a licorice representation of the protein bound to either Z-DNA or rGQ. Bonds and residue numbering are shown in the licorice view of each complex. (A) ZPF75 (UNIPROT: Q9NU63); (B) KLF2 (UNIPROT: Q9Y5W3); (C) ZSCAN4 (UNIPROT: Q8NAM6). All coordinates were assessed from https://www.uniprot.org/ on 18 May 2025. The Z-DNA sequence is d(CG)_4_ : d(GC)_4_. The rGQ sequence is r(AAGGGUUAGGGUUAGGGUUAGGGUU).

**Figure 2.**
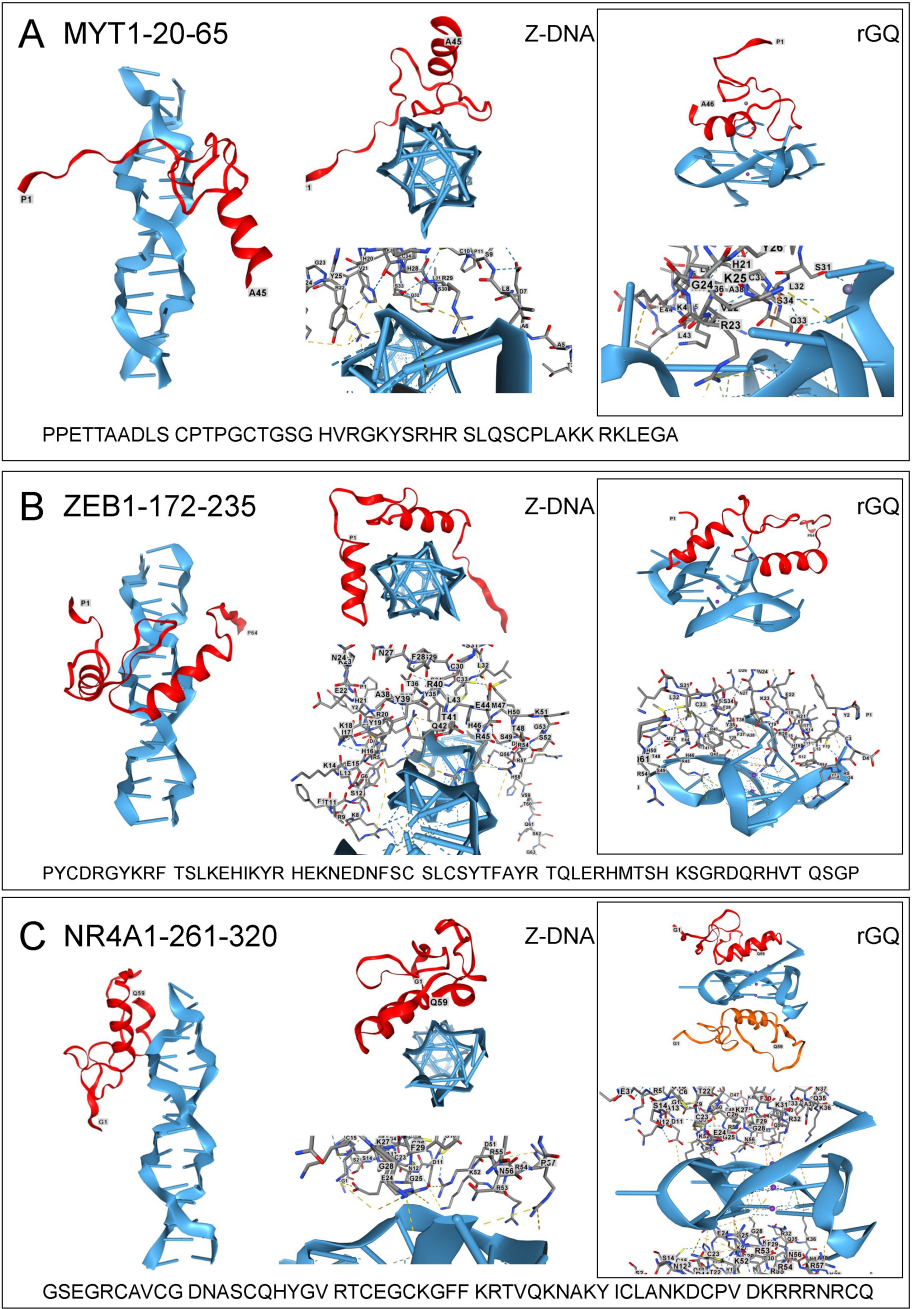
Docking of transcription factors to Z-DNA and RNA G-quadruplexes. Z-DNA and GQ are coloured blue, and the ZFP is ruby. Bonds and residue numbering are shown in the licorice view of each complex. (A) MYT1 (UNIPROT: Q01538); (B) ZEB1 (UNIPROT: P37275); (C) NR4A1 (UNIPROT: P22736). All coordinates were assessed from https://www.uniprot.org/ on 18 May 2025.

**Figure 3.**
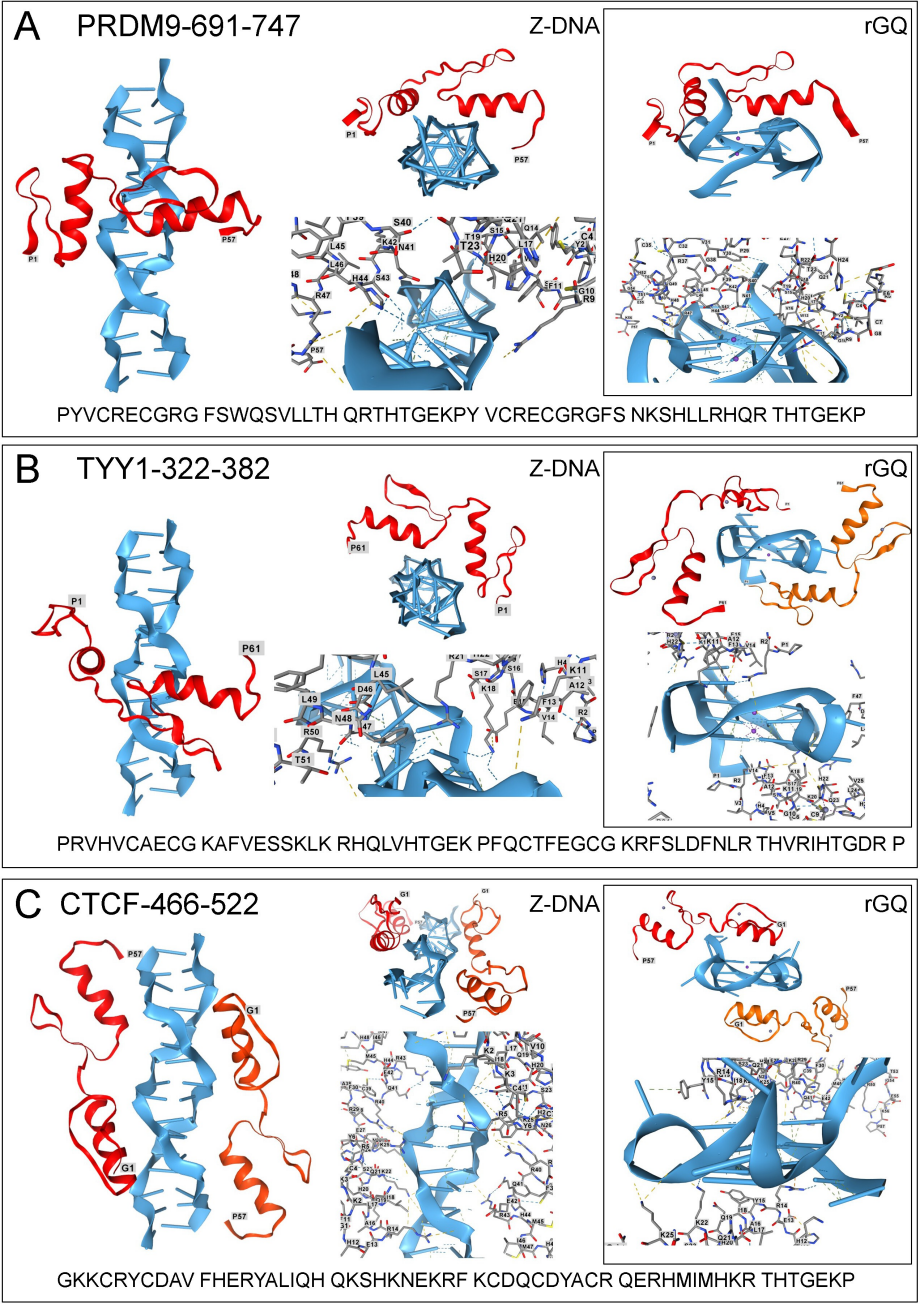
Docking of chromatin architectural proteins to Z-DNA and RNA G-quadruplexes. Z-DNA and GQ are coloured blue, and the ZFP is ruby. Bonds and residue numbering are shown in the licorice view of each complex. (A) PRDM9 (UNIPROT: Q9NQV7); (B) TYY1 (UNIPROT: P25490); (C) CTCF (UNIPROT: P49711). All coordinates were assessed from https://www.uniprot.org/ on 18 May 2025.

**Figure 4.**
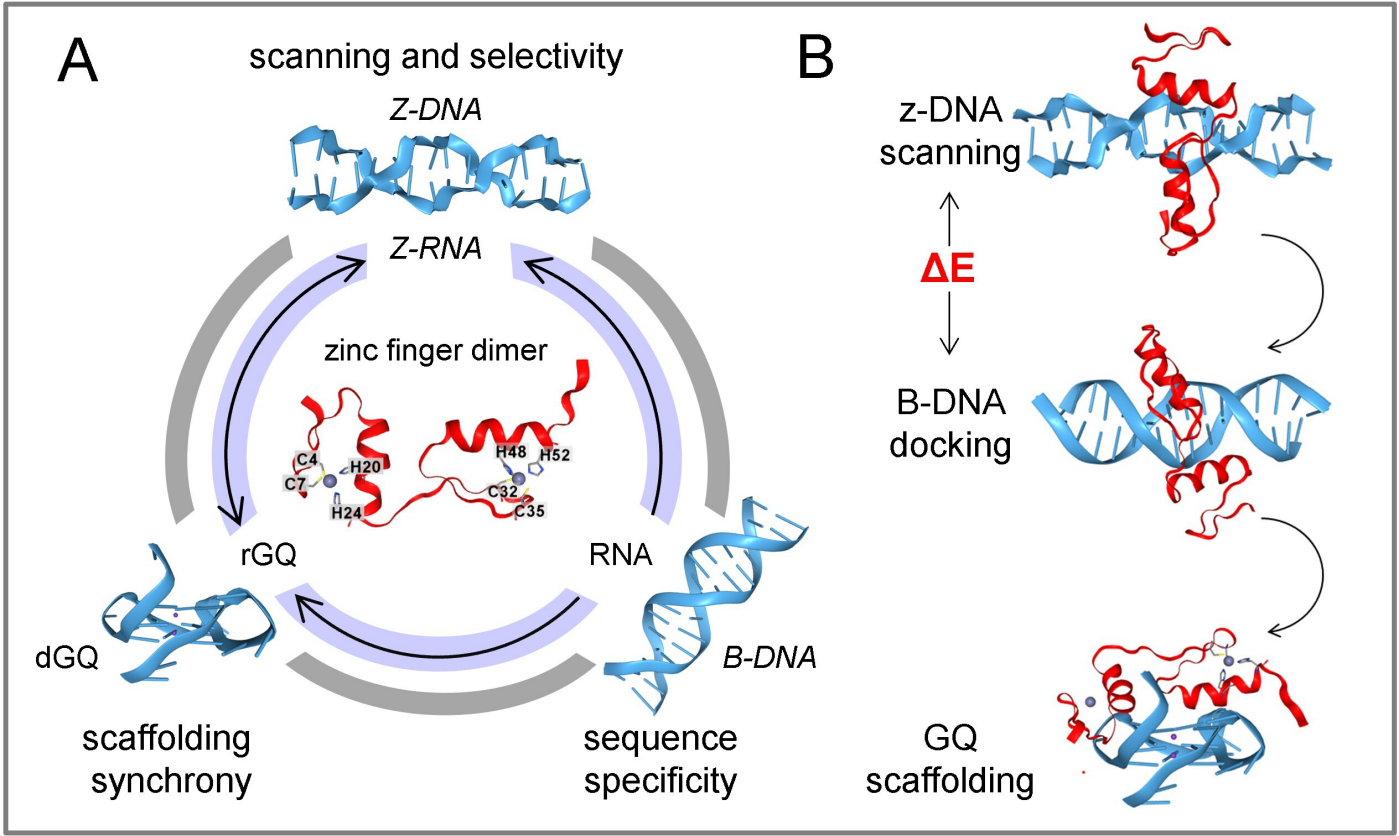
The interaction between ZFPs and flipons is dynamic. (A) Flipons adopt alternative conformations under physiological conditions. RNA (inner ring) and DNA (outer ring) can form the same alternative structures. Switching from one conformation to another can occur without strand cleavage or change in sequence. Some sequences can adopt both Z-DNA and G-quadruplex conformations. Zinc-finger domains are flexible and variable in their sequence preference, enabling them to interact with different flipon conformations. The relative affinities and avidity for different structures likely varies with the ZFP amino acid composition and the number of zinc finger clusters it contains. (B) The interactions between flipons and ZFP can occur in multiple ways. In this example, ZFP scans an open region of Z-DNA and then docks to a high-affinity B-DNA binding site. The energy gap between binding to Z-DNA and B-DNA optimizes the speed of scanning versus the stability of the interaction with B-DNA. Following the production of a transcript, the ZFP transfers a newly formed rGQ and scaffolds the assembly of an RNA processing complex.

To overcome this difficulty, a screen was developed using the AlphaFold algorithm that enabled visualization of protein interactions with flipon structures like Z-DNA and GQ. A mutation *in silico* could then help identify the key residues involved [44,132,139]. We have shown how these models validate with molecular dynamics simulations [132], and can provide key insights for wet-laboratory validation. Applying this approach reveals that ZFP dimers can bind Z-DNA and GQ-RNA (rGQ). These interactions are in addition to those involving sequence-specific interactions with B-DNA. The choice to use both DNA and RNA models was made to emphasize that structure-specific proteins usually can bind both nucleic acid types. The focus on GQ formed by RNA (rGQ) was for two reasons: rGQs are more frequent as they require only two guanosine tetrads to fold correctly, compared to the three for DNA-GQ (dGQ) [140]. Additionally, rGQs are involved in many aspects of RNA processing and stability [49,128,141–146]. Examples of KLFZP are presented in figure 1, transcription factors in figure 2 and chromatin architectural proteins in figure 3. The overall scheme of how ZFPs interact with different flipon conformations is presented in figure 4.

## 8. KLZP examples

ZNF57, a KZFP, is a primary regulator of imprinting in humans and mice that acts in combination with ZNF445 to ensure only one parental allele is transcribed [147,148]. The protein recognizes the persistent DNA methylation marks the zygote receives from a parent and promotes H3K9me3 modification at all but one of the known imprint control regions (ICRs) [149]. These marks are found on a d(TGCCGC) hexanucleotide present in all murine ICRs [150]. Interestingly, cytosine methylation promotes the flip to Z-DNA by Z-prone sequences [151]. This modification then potentially promotes the interaction of ZNF57 with Z-DNA.

The docking to Z-DNA of the first two ZNF57 ZF domains is shown in figure 1A. The flexibility of the linker between the dimers allows orientation of the α-helices to enable multiple contacts between the positively charged arginines. In the scenario given in figure 4, Z-DNA formation is promoted by methylation of parental DNA in the ICR. Initially, ZNF57 localizes to a region of Z-DNA formation, then scans for its cognate B-DNA sequence. If a site is found, ZNF57 locks onto the DNA. ZNF57 can also dock to a parallel rGQ formed by transcripts produced by neighbouring genes (figure 1A). These rGQs would compete with ZNF57 binding to DNA and limit the spread of heterochromatin to adjacent loci.

Other KLZPs have a more general role in regulating transcription in cells. One of these, Krüppel-like factor 2 (KLF2), has three C_2_H_2_-ZF domains. It belongs to the KLF/SP1 family of transcription factors with specificity for G-rich elements such as GC (GGGGCGGGG) and GT/CACC boxes (GGTGTGGGG), all of which can form Z-DNA and GQ [90,152,153]. The binding of various family members to GQ has been experimentally validated [120,138]. In AlphaFold models, KLF2 also engages both Z-DNA and rGQ when domains 1 and 2 are tested (figure 1B) or with domains 2 and 3 (data not shown). However, in models with all three domains, only binding to the B-DNA conformation was predicted. This finding is consistent with a model where recognition of a Z-DNA-containing region requires only two ZF domains. Once a cognate site is found, the engagement of the third domain flips the complex back to B-DNA and generates a high-affinity interaction.

ZFP can contain other effector domains besides KRAB. ZSCAN4 pairs a SCAN domain with four ZFs. The protein regulates embryonic stem cell potency and telomere length [154]. In two-cell zygotes, the ZFs bind to a TGCACAC motif found in the d(CA:TG) microsatellite repeat [155,156]. ZSCAN4 protects against double-strand breaks (DSBs) associated with this Z-prone motif. A similar outcome mediated by ZBTB43 is reported for d(CA:TG) repeats in sperm [157]. In the case of ZSCAN4, the set of ZF1, ZF2 and ZF3 docks to Z-DNA (figure 1C), while the set of ZF2, ZF3 and ZF4 flips the sequence to B-DNA (not shown). The three fingers also dock to the top and bottom of rGQ (figure 1C). Interestingly, a GU transcript produced by the d(TG) strand can also adopt a left-handed quadruplex fold [158].

## 9. Transcription and zinc-finger protein examples

An analysis of ZFP involved in differentiation and proliferation revealed that these domains interact with Z-DNA and rGQ (figure 2). Myelin transcription factor 1 (MYT1) regulates the formation of myelin and the proliferation and differentiation of oligodendrocytes [159]. The protein contains a transcriptional activation domain, a protein interaction domain and seven ZFs with different motifs. The first three ZFs are CCHC-type, with the first at portions 21−64 and the other two between amino acids 433 and 520. There are four C-terminal C_2_H_2_ ZF2 in positions 791−980 (UNIPROT: Q01538) [160]. Analysis of the first ZF revealed that this domain docks to Z-DNA and rGQ (figure 2A). The interaction involves residues outside of the classical α-helix.

ZEB1 promotes the transdifferentiation of epithelial to mesenchymal cells in cancers. The transition involves a feedback loop where ZEB1 represses expression of the microRNAs miR-200a, miR-200b, miR-200c, miR-141 and miR-429. In turn, the miR-200 family mRNAs inhibit ZEB1 protein translation [161]. This double-negative feedback loop allows a rapid switch from one phenotype to another [162]. When expressed, ZEB1 acts as a scaffold for different classes of cellular machinery. The interactions can activate or repress the transcription of many genes [163–165]. ZEB1 has seven C_2_H_2_ ZFs that lie in two clusters separated by a homoeobox domain. Both clusters contribute to ZEB1 function [166,167]. ZEB1 also engages Z-DNA and rGQ, as shown for the first two ZFs in figure 2B.

Another ZF transcription factor, nuclear receptor subfamily 4 group A member 1 (NR4A1, also known as NuR77 and NGFI-B), belongs to a family of orphan nuclear receptors and immediate early genes induced by multiple stressors that have pro-apoptotic cytoplasmic and anti-apoptotic nuclear roles [168]. Binding to DNA is activated by small molecules, with recognition of the nerve growth factor-induced-B response element (NBRE) d(AAAGGTCA) element [169]. Like other NR4A family members, NR4A1 can bind as a monomer, a homodimer or a heterodimer to single or tandem copies of NBRE. Three dimers can bind to the nuclear response element (NuRE) d(GATCCTAGTGATATTTACC TCCAAATGCCAGGA) [170,171]. NR4A1 contains two NR C4-type ZFs where zinc is coordinated only by cysteines. The first ZF can dock as a monomer to Z-DNA (figure 2C). Adding a second monomer to the model flips the sequence to B-DNA, indicating that Z-DNA may help find a cognate binding site, but high-affinity binding requires engagement of two ZFP (not shown). The protein monomer binds to rGQ when either one or two copies are included in the model (figure 2C).

## 10. Chromatin and zinc-finger protein examples

The PRDF1-RIZ (PR) domain containing protein 9 (PRDM9, also known as Meisetz) is a subtype of the SET domain (Su(var)3-9, enhancer-of-zeste and trithorax) family of histone methyltransferases. The histone H3(K4) trimethylation produced by PDRM9 leads to the transcriptional activation of genes. The outcome depends on the PDRM9 KRAB domain, which is likely a precursor of other KRAB domains that later evolved suppressive roles due to amino acid deletions [172].

PRDM9 is expressed in the testis and ovaries [173]. The gene is active during meiosis I and regulates recombination events by marking DNA for double-strand breaks at locations targeted by the ZF domain [174]. Mouse PRDM9 knockout causes sterility in both sexes due to severe impairment of the double-stranded break repair pathways in meiosis I [173]. Male sterility also results when certain mouse strains with different PRDM9 alleles are interbred, producing male-specific hybrid dysgenesis. In this situation, PRDM9 acts as a speciation gene: the lack of fertile offspring reproductively isolates each strain [175].

In mouse crosses that produce hybrid sterility, the observed number of synaptonemal complexes involved in recombination are greatly diminished [176]. The results suggest that the PRDM9 alleles interfere with the DSB-mediated pairing of the non-homologous chromosomes necessary to form the bridges essential to their independent segregation during meiosis. Comparison of PRDM9 sequences across 19 mammalian genomes shows rapid divergence in the DNA residues contacted by the ZF domains. Positions −1, 2, 3 and 6 of each ZF show positive selection in humans and chimpanzees. In contrast, ZF within a species shows sequence convergence [174,177,178]. The count of ZF domains in PBRM9 orthologues varies between 6 and 19. Interestingly, the allele repertoire for each primate species is predicted to bind distinct motifs with little to no overlap between species [174].

Structural studies of the ZF8-ZF9 array from human PRDM9 confirm recognition by this construct of a consensus motif d(NCCNCCNTNNCCNCN)n derived from hotspot meiotic recombination hotspots [179]. Conserved histidine residues at position −4 or conserved arginine residues at position −1 of ZnF8, ZnF9, ZnF11 recognize d(C:G) base pairs. The asparagine at position −4 of ZnF10 recognized a T:A base pair. These interactions involved bidentate contacts. ZF12 was not mappable within these structures. In structures formed with different sequences, the amino contacts varied to intimately fit the ZF array to the DNA target. Indeed, PRDM9 bound with similar affinity to the THE1B and MSTM1b-1 sequences, even though these differ at nine out of 15 base pairs in the five triplets bound.

Hybrid sterility in mice may reflect the different sequence specificity of each PRDM9 allele. However, a genome-wide mapping of human recombination hotspots revealed that sequence variation explains less than 44% of the variation. The results suggest that factors such as binding site accessibility must strongly affect DSB initiation frequency [180]. Other studies show a bias during meiosis for pairing a homologous chromosome with the non-homologous chromosome. This outcome depends on masking sites on the homologous chromosome by cohesin. This coating prevents the use of a homologous site as a template to guide DSB repair [181]. In the simplest case, the positioning of cohesins by PDRM9 alleles on each parental chromosome differs between strains with hybrid sterility. The approximately 50−300 base-pair open sites required for DSB repair no longer align [182]. This outcome may reflect differences in the sequence specificity of the PRDM9 allele. Alternatively, each PRDM9 allele localizes to different sites of Z-DNA formation in each parental genome.

Overall, human PRDM9 has 14 C_2_H_2_-ZF domains. The binding of ZF8 and ZF9 from PRDM9 to Z-DNA and rGQ is shown in figure 3A. Recognition of these flipon conformations potentially allows PRDM9 to position the cohesins essential for pairing non-homologous chromosomes during meiosis. This formation of synaptonemal complexes involving Z-DNA may be favoured in sperm, where histones are sequentially replaced with protamines, leading to a more open chromatin structure [183–187]. Notably, the PRDM9 locus is itself a recombination hotspot [188]. Inspection of the PRDM9 locus reveals the presence of many d(CA)_*n*_ Z-prone sequences. A subset of these repeats encodes the terminal histidine in the ZF domain (GenBank Accession DQ388610.1, accessed 15 May 2025) [88]. Similarly, other Z-DNA-forming sequences may be associated with increased recombination throughout the genome. Z-DNA-forming sequences are indeed associated with the structural instability of the coding regions that leads to Mendelian diseases [21].

ZFPs also bridge different chromosomal regions in somatic cells. Yin Yang 1 (YY1) is a ubiquitously expressed gene identified initially as a transcriptional modulator. YY1 has four ZF1 fused to a variety of transcriptional activator and repressor domains. The complexes they assemble can acetylate histones or form repressive structures. YY1 also interacts with various chromatin remodellers [189]. In these different roles, YY1 dimers localize enhancers to promoter sequences in a context-specific manner to regulate gene expression [190]. Acting in concert with the CCCTC-binding factor (CTCF), YY1 orchestrates the reorganization of chromatin loops during neural progenitor cell differentiation [191]. YY1 also plays an essential role in regulating gene expression at the morula stage of embryonic development [192].

YY1 has four ZF domains. YY1 homodimers bind a d(CAT) core in a variable sequence context of d(C/g/a)(G/t)(C/t/a)CATN(T/aXT/g/c), where the capitalized letter indicates the preferred base [193,194]. All three ZF pairs bind Z-DNA. The model for ZF2 and ZF3 is shown in figure 3B. Previous studies have revealed that YY1 binds to GQ [120,195] as also shown in figure 3B. The modelling of ZF1 and ZF2 with GQ, ZF3, and ZF4 reveals extensive contacts consistent with the experimental data. Interestingly, docking of ZF2 and ZF3 to GQ is best modelled with two copies of the domain (figure 3B), suggesting that in some situations, GQ formation will bridge different YY1 complexes. Alternatively, YY1 could bind to one face of a GQ, with a different GQ ligand, such as a helicase or chromatin modification complex, engaging the other side.

The interaction between YY1 and CTCF is important for insulating actively transcribed genes from neighbouring regions. The boundaries formed help define topologically associated domains (TADs) containing DNA loops [196]. As a result, the supercoiling generated within the loops is constrained from spreading to an adjacent locus. This energy is then available to power the transactions within the loop that enable the transcription and processing of pre-mRNAs. The organization of TADs varies with cell type and development stage [197–202]. Other functions for this protein may exist as CTCF binds sequences besides the canonical motif, both in tumours [203] and in cultured cortical neurons [204]. These variant sequences may account for up to 22% of CTCF-bound sites, and are found in the promoter regions where CTCF localization also changes during differentiation [204].

The core CTCF binding motif is set by the nucleotide specificity of the first seven zinc fingers, with ZF1 docking at the 3′ end of the nucleotide sequence. CTCF binding is diminished by DNA methylation [205–207]. The orientation of CTCF helps define TAD boundaries and regulates loop formation within these domains. The loops are created by extruding DNA through a ring composed of three cohesin proteins. Contacts between the CTCF N-terminus and cohesin prevent the threading of DNA movement through the ring. In contrast, the interactions of the C-terminus with cohesin either have no effect or may accelerate DNA movement [208,209]. Convergent CTCF binding sites then act as gates. By pointing the N-terminus of each CTCF monomer towards the other, the gates confine the direction in which cohesin moves the DNA segment between them. The physical interaction of CTCF with cohesin involves the radiation-sensitive mutant 21 (RAD21) protein, which is involved in DNA double-strand break repair [210].

Interestingly, the CTCF interaction with DNA strengthens as active processes inside the loop increase the DNA tension [209]. Such stresses can also induce Z-flipon sequences to form Z-DNA [211,212]. Within loops, the movements of cohesin can be further limited by R-loops, where an RNA:DNA hybrid arising from transcription can expel the other DNA strand from the helix. The single-stranded DNA can then form a GQ if it contains a G-flipon sequence [213–217]. Notably, both the supercoiling and R-loop formation are dependent on transcription. Indeed, CTCF binding at low-affinity sites in DNA is disrupted by RNA polymerase inhibitors [218].

CTCF finds cognate binding sites by scanning along DNA [209]. In the available structures, ZF3-7 and ZF9-11 recognize sequences by making base contact. ZF8 is bound nearly parallel to the DNA axis and only contacts the phosphate backbone. The potential of CTCF to interact with Z-DNA and GQ is confirmed by modelling. Interestingly, the ZF8 and ZF9 dimer docks parallel the Z-DNA axis, with the same 3′–5′ orientation as ZF8 binding to B-DNA. The binding of two of the ZF8-ZF9 monomers to Z-DNA is mediated by phosphate and base-specific contacts (figure 3C). Two ZF8-ZF9 monomers also dock to rGQ, one to each face of the quadruplex.

The binding of CTCF to RNA through ZF1 and ZF10 has been experimentally confirmed. Mutations to ZF1 and ZF10 diminish CTCF’s ability to bind RNA without impacting interactions with DNA. Deletion of ZF1 leads to a reduced presence of CTCF at promoters, as does transcriptional inhibition. Deletion of ZF10 affects targeting of CTCF to intronic and intergenic sites. Both mutations alter chromatin structure [218]. CTCF also contacts RNA through amino acids that lie immediately carboxy-terminal to ZF11 (cRBS). The 36 amino acids are in an intrinsically disordered region of the protein. Deleting the cRBS affects the ability of CTCF to self-oligomerize. The mutation also alters around half of the chromatin loops detected in unperturbed mouse embryonic stem cells, leading to dysregulated gene expression [219]. Modelling of the cRBS in conjunction with ZF11 shows that each region binds to opposite sides of an rGQ (not shown). The interaction with the rGQ may help stabilize condensates in chromatin regions where G-flipons are transcribed.

## 11. New approaches to identify proteins that bind alternative flipon conformations

The recognition of flipon structures by ZFP expands the genetic lexicon. The findings help resolve questions about the biological relevance of these alternative structures. The results also help explain the difficulties in isolating Z-DNA-specific binding proteins: the problem arises because each ZF domain can potentially bind Z-DNA, B-DNA and GQ. The ability to detect each type of interaction depends on the experimental design. The usual experimental approaches only capture the low-energy B-DNA state. Often, a B-DNA competitor is used in assays as it is assumed that the interactions with different DNA structures are mediated through separate domains. Failure of B-DNA to compete with the docking of a protein to an alternative conformation then confirmed the specificity of the proposed interaction. While this approach worked for the discovery of the Zα because of the slow off-rate of Zα from Z-DNA [220], it is not generalizable. Other methods are necessary. For example, the availability of stable GQ reagents facilitates the detection of GQ binding proteins. Modified probes that cannot form the Hoogsteen hydrogen bonds required to stabilize a G4-tetrad are appropriate specificity controls.

A rationale now exists for extending experimental methods to study the interaction between ZFP and the more dynamic Z-DNA conformation. Approaches using fluorescent resonance energy transfer and single-molecule atomic tweezers have previously been deployed to study B–Z and A–Z transitions [130,211,212,221–223]. These methods could be adapted to study the interactions of ZFPs with Z-DNA. The studies will also allow evaluation of how base-specific contacts with the left-handed helix affect ZFP function. It may be possible to produce ZFP variants that recognize a particular Z-DNA sequence motif to target a clinically relevant subset of canonical B-DNA binding sites. It is also likely that C_3_H_1_-type ZFPs, like the regnase and roquin families [224], and proteins that bind CpG dinucleotides, will also be characterized as ZNA binders and contribute to the regulation of innate immune response driven by Z-RNAs.

## 12. The coevolution of flipons and zinc-finger proteins

The findings overall are consistent with the early evolution of both flipons and ZFP in prebiotic systems. They build on the idea that self-replicating molecules composed of flipons and metal-bound dipeptides lead to the evolution of the modern-day genetic code [88]. During evolution, ZFPs have evolved from a structure-based form of genetic encoding to one based on sequence. This B-DNA base-specific recognition code is built on the earlier one. Scanning for an alternative structure formed in transcriptionally active regions in larger genomes reduces the search space for a high-affinity binding site.

The simple design and flexibility of ZFP oligomers enabled recognition of different flipon sequences that facilitate the assembly of more complex chemistries. In this scheme, the interaction strength with different flipon conformations could be modulated by adducts that affect zinc binding to its coordination site. Such examples include the redox-sensitive formation of cysteine adducts during metabolic stress and histidine methylation of metal coordination sites that reduce the rigidity of the fold [225].

ZFPs are usually composed of many ZF clusters. This evolutionary embellishment helps counter TE expansion and escape from surveillance. One challenge is to prevent the sequestration of ZFP by related B-DNA sequences elsewhere in the genome that pose no threat to the host. The issue is exacerbated by the capability of tandem ZF domains to recognize many alternative sequences, or just a few very long ones [83]. How then does one rapidly target those actively transcribing EREs that constitute a threat to their host? A fast-scanning mechanism is required that is at odds with the high-affinity binding of ZFP to its cognate sequence. The problem is known as the speed–stability paradox [226,227]. A conformational switch between scanning and recognition modes offers a potential resolution of this conundrum. The energy gap between the two states enables a fast search for a high-affinity cognate site with fast dissociation when one is not present [226]. The energy stored in Z-DNA is then available to power further assembly of the protein complex at this site following sequence-specific docking of the ZFP to DNA.

Viewed from this perspective, the binding of ZFPs to TE is not solely determined by whether they are transposition competent, but rather by whether they are actively transcribed. In the case of ERV, ZFP only localizes to sequences that form an alternative structure. They no longer remain targets if they cannot flip to an alternative conformation—cycling between alternative conformations is a necessary part of transcriptional reinitiation for many genes [50,228]. The TE flipon sequences may mutate, undergo epigenetic modification or be buried in heterochromatin. Such processes also enable infectious retroviruses to enter a latent phase. Over time, ERVs often undergo recombination and survive as solo LTRs. These with active flipons undergo exaptation to regulate cellular genes. They do so by creating open regions of chromatin that incorporate B-DNA binding sites for TFs. Earlier in their history, this ability to develop open areas and engage TFs allowed for their expression and retrotransposition throughout the genome. Each attack by a new family of retrotransposons enables this cycle to repeat and to create new evolutionary opportunities.

The scan and dock mechanism is well known for ensuring the binding of tRNAs to cognate codons at the A-site of the ribosome. This process utilizes a kinetic proofreading mechanism [229]. The on-rate of binding to the ribosomal acceptor site is similar for all tRNAs, but only cognate and near-cognate tRNAs have a slow off-rate. The energy released by GTP hydrolysis can lead to further stabilization of the cognate tRNA interaction and is required for high-fidelity translation [230]. The scan and dock mechanism provides a similar solution to allow ZFP to scan for its cognate sequence rapidly, and then transition to a high-affinity interaction when a site is discovered. The process enables testing of multiple zinc fingers within the protein to see whether any is specific for a sequence in a particular region of DNA. In principle, this design allows for duplication and diversification of each zinc finger domain in much the same way as Ohno proposed for gene-based evolution. The gains and losses of ZF domains in a protein lead to the evolutionary selection of variants that improve the propagation of a species [231]. The biological outcome of engaging a particular ZF domain depends on the protein effector domain activated by the interaction. Some ZFPs, like ZEB1, can assemble context-dependent complexes that either activate or repress gene activation to protect the cell. Others, like ZSCAN4, can promote nucleosome assembly to store energy by coiling DNA around histones. Ejection of the nucleosome at a later time then releases the energy to power other transctions [232].

ZFPs also show other cycles of adaptation. Increasing the number of ZF domains in a protein allows the clusters to span larger physical distances. This extra reach can augment the sequential scanning of widely separated DNA segments by the process of stepping over nucleosomes [233] or even enable a jump from one chromosome to another [234]. This random walk increases the probability of finding a high-affinity B-DNA binding site. Once the protein is docked, the search can continue to find another cognate binding site that involves a different ZF domain. The longer length of some ZF tandem arrays allows for scanning a larger area of the nucleus. Binding the ZFP to separate sites enables bridging of physically distant chromosomal regions and the approximation of widely separated promoters and enhancers. The search is facilitated by the open chromatin regions created by both long noncoding RNAs (lncRNAs) and small RNAs. The recognition by ZFP of those rGQ formed by lncRNAs is part of this process and provides another way of forming condensates that anchor the connections between different chromosomal segments. Overall, the generation and natural selection of novel ZF tandem arrays contribute to phenotypic diversity without necessitating the modification of those ZF domains currently depends upon.

The interaction of ZFP with GQ enhances other aspects of cellular function. In enhancer–promoter condensates, the docking of ZFP to rGQ ensures the retention of sequence-specific TFs released as promoters are reset following a transcriptional burst [50]. The TFs then bind to rGQ formed in the RNA cloud surrounding the promoter. The binding of TF to both a GQ and a cognate B-binding site also enables regulation of transcriptional bursting. The process depends on RNA polymerase pausing 50−100 bases downstream following production of a short RNA transcript. The paused polymerase is released only under specific contexts. Pausing is associated with the formation of a GQ scaffold that enables the assembly and licensing of an RNA–polymerase complex complete with the factors requred for transcriptional processivity and processing of the RNA transcript. Once RNA elongation is initiated, the promoter is reset with GQs then resolved by helicases. GQ-bound TFs are released and free to re-engage their cognate binding sites. The load, wait, and fire cycle then reinitiates. This process enables coordinated responses, ensuring that they are triggered under specific circumstances and in synchronization with other responses. GQs are also involved in the processing of nascent transcripts. They form GQ-dependent scaffolds that localize the complexes necessary to complete the co-transcriptional modification, editing and splicing of RNA [50].

The different forms of interaction between flipons and ZFP are illustrated in figure 4A. The illustration emphasizes the highly dynamic modes of ZFP interaction with flipons. The flexibility of ZF loops enables docking of ZFPs to different flipon conformations. Contacts with both the phosphate backbone and the base pairs are involved with those to B-DNA, enabling high-affinity interactions, but with faster off-rates. The various DNA and RNA flipon folds are highlighted in the figure, with some sequences initiating different outcomes by flipping either to a GQ or to ZNA.

The interactions between flipons and ZFPs can actualize many different downstream designs. In the example shown in figure 4, a ZFP scans Z-DNA to discover a cognate B-DNA binding site. The energy stored by Z-DNA is then available to power the assembly of the protein complex scaffolded by the ZFP. The GQ formed by the transcripts then promotes the transfer of the ZFP from DNA to RNA, where the protein can scaffold the assembly of the RNA processing machinery. Overall, it is likely that many more proteins bind alternative flipon conformations than is currently appreciated. The methods described here are useful for screening ZFPs and other nucleic acid binding proteins to discover interactions with alternative DNA and RNA structures that have previouly escaped notice.

## 13. Conclusion

The entanglement of flipons with ZFP underlies a robust strategy to counter the threat posed by active and evolving retrotransposons. The system depends on two alternative forms of encoding genetic information. Flipons flag transcriptionally active TE by changing conformation, thereby facilitating the discovery by ZFPs of retroelement B-DNA sequences for which they have high affinity. The threats posed by transposons and other intracellular pathogens drive the coevolution of flipons and ZFPs. The exaptation of TE encoded flipons to control gene expression and transcript processing engenders many roles for ZFP coding variants in the generation of phenotypic diversity.

## Data Availability

Protein database files are provided for the structures shown in figures 1–3. Supplementary material is available online [235].
